# Comparison of vancomycin assays in patients undergoing hemodialysis

**DOI:** 10.1016/j.bjid.2024.103869

**Published:** 2024-09-16

**Authors:** Letícia Scribel, Aline Galiotto, Isadora de Souza Rodrigues, Roberta Hahn, Rafael Linden, Alexandre P. Zavascki

**Affiliations:** a Universidade Federal do Rio Grande do Sul, Faculdade de Medicina, Programa de Pós-Graduação em Ciências Médicas, Porto Alegre, RS, Brazil; b Instituto Federal de Educação, Ciência e Tecnologia do Rio Grande do Sul, Campus Porto Alegre, RS, Brazil; c Hospital Moinhos de Vento, Serviço de Farmácia, Porto Alegre, RS, Brazil; d Universidade Feevale, Instituto de Ciências da Saúde, Laboratório de Toxicologia, Novo Hamburgo, RS, Brazil; e Infectious Diseases and Infection Control Service, Hospital Moinhos de Vento, Porto Alegre, RS, Brazil; f Infectious Diseases Service, Hospital de Clínicas de Porto Alegre, Porto Alegre, RS, Brazil; g Department of Internal Medicine, Universidade Federal do Rio Grande do Sul, Porto Alegre, RS, Brazil

**Keywords:** CMIA, Hemodialysis, KIMS, LC-MS/MS, Vancomycin

## Abstract

Vancomycin is a glycopeptide antibiotic mainly excreted by glomerular filtration. Therefore, patients undergoing hemodialysis tend to accumulate its crystalline degradation product, which has been associated with cross-reaction in commercial immunoassays. The aim of this study was to assess the performance of two commercial immunoassays for measuring vancomycin levels in patients undergoing hemodialysis. This method-comparison study enrolled patients undergoing hemodialysis at two hospitals in Porto Alegre, Brazil. Vancomycin serum concentrations measured by Chemiluminescent Microparticle Assay (CMIA) and measured by Kinetic Interaction of Microparticles in Solution (KIMS) were compared with Liquid Chromatography coupled with Tandem Mass Spectrometry (LC-MS/MS). A total of 64 samples from 42 patients and 54 samples from 23 patients were included in CMIA and KIMS groups. Both measurements were highly correlated with LC-MS/MS, with Spearman rank correlation coefficient *r* = 0.840 (*p* < 0.001) and *r* = 0.926 (*p* < 0.001), respectively. No deviation of linearity was observed (*p* = 0.81 and *p* = 0.49, respectively). The mean difference between CMIA and LC-MS/MS was -1.19 μg/mL and between KIMS and LC-MS/MS was -2.28 μg/mL. LC-MS/MS measured levels were, on average, 2.64 % higher than CMIA and 8.81 % higher than KIMS. CMIA and KIMS revealed accurate commercial methods to measure vancomycin serum concentrations in patients undergoing hemodialysis.

## Introduction

Vancomycin is an antibiotic glycopeptide discovered in the 1950s and approved by Food and Drug Administration (FDA) to treat severe infections caused by gram-positive bacteria, especially methicillin-resistant *Staphylococcus aureus (*MRSA*)*.^1^,^2^ Despite more than 60 years of clinical use, questions still need to be answered regarding strategies to individualize therapy, minimize toxicity, and prevent increased bacterial resistance.^3^

Vancomycin therapeutic level monitoring is highly recommended due to its narrow therapeutic index,^4^ wide interpatient and intrapatient pharmacokinetic variability along with exposure-dependent nephrotoxicity.^5^ Therapeutic Drug Monitoring (TDM) rests on pharmacokinetic/pharmacodynamic (PK/PD) studies, which embrace three PK/PD indices: ratio of maximum concentration to minimum inhibitory concentration (MIC), ratio of area under the concentration-time curve to MIC and time concentration above MIC.^6^ Vancomycin antimicrobial effect is dependent on the ratio of the area under the plasma concentration-time curve during 24 h (AUC_0–24_) over the minimum inhibitory concentration by broth microdilution (AUC_0–24_/MIC_BMD_), and a value between 400 and 600 mg.hour/L is indicated as the best target to treat MRSA invasive infections.^7^

As vancomycin quantification is mandatory for TDM, robust assays are required to ensure adequate, effective, and safe serum concentrations.^8^ Although immunoassays are the most commonly used for vancomycin measurement in clinical laboratories, chromatography methods coupled with tandem mass spectrometry are considered the gold standard for their high specificity and reproducibility.^9^ Even though immunoassays enable simple and fast measurements by consolidated analytical systems and commercial kits, inconsistent accuracy and precision may occur due to interference of cross-reacting substances, such as the biologically inactive vancomycin crystalline degradation product, CDP-1.^10^

Vancomycin is mainly excreted by glomerular filtration. Serum half-life in patients with normal renal function is approximately 6 h, however, renal impaired patients have a prolonged half-life of up to 200 h.^4^,^11^ The prolonged exposure of the drug to body temperatures allows CDP-1 to accumulate.^12^ Since CDP-1 has chemical structures similar to vancomycin, there are several reports about cross-reaction with anti-vancomycin antibodies in non-specific immunoassays, leading to falsely elevated vancomycin levels.^12^, ^13^, ^14^, ^15^, ^16^

Infectious diseases are the second leading cause of death in patients with chronic kidney disease undergoing hemodialysis (HD), preceded only by cardiovascular disorders.^11^ The skin disruption by HD vascular access is the main route of infection, leading to bacteremia commonly caused by MRSA.^11^ Vancomycin is widely prescribed for patients undergoing HD, and TDM is recommended by the latest consensus guideline published by Ryback et al.^17^ Therefore, it is crucial to elucidate if currently available immunoassays are susceptible to CDP-1 interference.

This study aims to compare two immunoassays, Chemiluminescent Microparticle Assay (CMIA) and Kinetic Interaction of Microparticles in Solution (KIMS), with Liquid Chromatography coupled with Tandem Mass Spectrometry (LC-MS/MS) for vancomycin measurements from patients undergoing HD to avoid dose adjustment based on falsely elevated vancomycin serum levels.

## Materials and methods

### Patients, samples, and immunoassay methods

This study involved two Hospitals from Porto Alegre City, Rio Grande do Sul (RS), Brazil. From December 2020 to June 2021, a total of 64 samples analyzed for vancomycin TDM by CMIA (ARCHITECT iSystem, Abott™) from 42 patients undergoing HD at Hospital de Clínicas de Porto Alegre (HCPA) were included in CMIA cohort. From October 2021 and February 2022, 54 samples analyzed for vancomycin TDM by KIMS (Cobas® 6000, c501 module, Roche/Hitachi) from 23 patients undergoing HD at Moinhos de Vento Hospital (HMV) were included in KIMS cohort. The sample size was estimated based on an Clinical Laboratory Standard Institute (CLSI) analytical guideline,^18^ which suggests the analysis of at least 40 samples for method comparison studies. Calibration, quality control, and patient samples were routinely processed according to the manufacturer's instructions.

CMIA immunoassay is a competitive chemiluminescent immunoassay. Vancomycin in the sample competes with acridinium ester-labeled vancomycin for a limited amount of anti-vancomycin monoclonal mouse antibody covalently bound to paramagnetic particles. The resulting chemiluminescence is inversely proportional to the vancomycin concentration in the sample. KIMS immunoassay is based on the kinetic interaction of microparticles in a solution. Vancomycin in the sample competes with a vancomycin conjugate for anti-vancomycin antibodies, covalently bound to microparticles. The kinetic interaction of the microparticles is inversely proportional to the concentration of vancomycin in the sample.^19^

According to the kit label, vancomycin CMIA assay range is 0.24 μg/mL to 100.00 μg/mL. The measurement was developed to have a precision of ≤ 10 % total CV (range 3.1 % to 6.2 %) and a mean recovery of 100 ± 10 %. The sensitivity, defined as the Limit of Detection (LoD), was ≤ 2.0 μg/mL. According to KIMS manufacturer, vancomycin measuring range is 4.0 to 80.0 µg/mL, precision of ≤ 10 % total CV (range 2.3 % to 8.2 %) LoD equal to 1.5 µg/mL (1.04 µmoL/L).

All samples were collected up to one hour before vancomycin administration. The serum samples were collected in gel-enhancing serum tubes, centrifuged at 18,000 rpm for 15 min, and were immediately analyzed. The remaining material was stored at −20 °C for up to 15 days for further analysis by LC–MS/MS at the Laboratory of Analytical Toxicology, Feevale University, Novo Hamburgo, RS, Brazil.

### Liquid chromatography coupled with tandem mass spectrometry method

Stock solutions were prepared by dissolving vancomycin (vancomycin hydrochloride, Sigma Aldrich – Milan, Italy) in ultra-pure water (6 mg/mL) and atenolol-D7 (Sigma Aldrich, Saint Louis, USA) in acetonitrile (200 mg/mL). Atenolol-D_7_ (9 mg/mL) was used as Internal Standard (IS), prepared from stock solution.

Calibrators and Quality Control (QC) were obtained by spiking vancomycin stock solution. A six-point calibration curve was created from calibrator standards prepared by adding vancomycin to human plasma to yield concentrations of 2.5, 5.0, 10, 25, 50, and 75 μg/mL. This calibration curve covers the expected concentrations in clinical samples. QC was prepared at a concentration of 15 μg/mL. A volume of 50 µL of each plasma sample, calibrator, and QC was mixed with 100 µL atenolol-D7 (9 mg/mL in acetonitrile), followed by vortex-mixing and centrifugation. An aliquot of 100 µL of the supernatant was mixed with 600 µL of ultra-pure water and 100 µL of dichloromethane, followed by vortex-mixing and centrifugation. An aliquot of 100 µL of the supernatant was transferred into a new vial.

The LC-MS/MS system consisted of an Acquity I-Class system connected to a XevoTQS-micro triple quadrupole mass spectrometer, both acquired from Waters Technologies (Milford, USA). Chromatographic separation was performed with an Acquity BEH C8 column (100 × 2.1 mm, p.d. 1.7 μm), also acquired from Waters. The column temperature was 35 °C and the injection volume was 1 μL. Mobile phases were 0.1 % formic acid in water (A) and 0.1 % formic acid in acetonitrile (B), eluted at a flow rate of 0.3 mL/min. The total run time was 6 min. MS conditions were: ionization in electrospray positive ion mode and capillary voltage 1.50 kV. The desolvation temperature was 400 °C, the desolvat002Dion flow was 600 L/h and the cone flow was 40 L/h. The monitored multiple reaction transitions (*m/z*) were: vancomycin 725.5→144 for quantification and 725→100 for qualification and atenolol-D7 274→145 for quantification. Vancomycin and atenolol-D_7_ retention time was 2.36 and 2.35 min, respectively. The assay was validated according to international guidelines.^19^ Intra-assay precision is 3.12 %‒4.81 %, inter-assay precision is 2.65 %‒4.69 %, and accuracy is 98.4 %‒99.6 %.

### Method comparison

The agreement between serum vancomycin concentrations measured by each immunoassay (CMIA and KIMS) and LC-MS/MS was performed using nonparametric Passing-Bablok regression analysis, with 95 % Confidence Intervals (95 % CI) calculated for the slope and intercept. The Bland-Altman plots were used to assess the relative differences between CMIA, KIMS, and LC-MS/MS methods by plotting the percentage differences against the mean vancomycin value for each immunoassay and LC-MS/MS. The mean relative differences and the 1.96 Standard Deviations (SD) of the differences were calculated, and values within 1.96 SD were considered acceptable. The discordance of CMIA and KIMS compared to LC-MS/MS was reported as percentage. All the statistical analyses have been performed with Medcalc software (MedCalc Software Ostend, Belgium).

Incurred Sample Reanalysis (ISR) criterion, described in the FDA Bioanalytical Method Validation Guidance for Industry, was used to verify the reliability of the sample analyte concentrations and to critically support the precision and accuracy measurements established with the quality controls. The percentage differences in the results between the reference method and the tested method were determined with the following equation: (TestedMethod−ReferenceMethod)/Mean*100.

The difference between the two values obtained should be within 20 % of the mean for at least 67 % of the samples.^20^

## Results

The CMIA evaluation consisted of 64 serum samples, obtained from 42 patients, measured for vancomycin concentration as part of the TDM routine. Of the 42 patients, 28 (66.7 %) were men, and the mean age was 58 years (range: 30–84 years); 38 were receiving continuous renal replacement therapy (CRRT), 2 were receiving intermittent HD (IHD), 1 patient changed from CRRT to IHD and 1 was receiving peritoneal dialysis. The KIMS assessment consisted of 54 samples from 23 patients, measured for vancomycin concentration as part of the TDM routine. There were 17 (73.9 %) men, and the mean age was 75 years (range: 34–89 years); 14 were receiving CRRT, 5 were receiving IHD, 1 patient changed from CRRT to IHD and 3 were receiving peritoneal dialysis. The frequency of prescribed vancomycin doses for studied samples in the CMIA and KIMS cohorts is listed in Table 1. Vancomycin doses were administered every 12 h in 73 % and 63 % and every 24 h in 22 % and 33 % of CMIA and KIMS cohorts, respectively. In both CMIA and KIMS evaluations, all patients used high-flux membrane dialysis, except one who was on peritoneal dialysis.

**Table 1:** Frequency of prescribed vancomycin doses for studied samples according to the method used to respectively measure serum concentrations.

CMIA cohort prescribed vancomycin doses	Frequency	KIMS cohort prescribed vancomycin doses	Frequency
1000 mg 12/12h	16	1000 mg 12/12h	17
750 mg 12/12h	11	1000 mg once a day	10
1000 mg once a day	9	500 mg 12/12h	10
1500 mg 12/12h	6	500 mg once a day	6
500 mg 12/12h	6	750 mg 12/12h	6
1250 mg 12/12h	5	500 mg, oral route^a^, 6/6h	2
1500 mg once a day	2	1250 mg 12/12h	1
250 mg 12/12h	2	750 mg once a day	1
500 mg once a day	2	1500 mg once a day	1
24 h according medical order	2	‒	‒
2000 mg 12/12h	1	‒	‒
500 mg 8/8h	1	‒	‒
750 mg once a day	1	‒	‒

CMIA, Chemiluminescent Microparticle Assay; KIMS, Kinetic Interaction of Microparticles in Solution.

a One patient also received vancomycin by oral route.

**Fig. 1:**
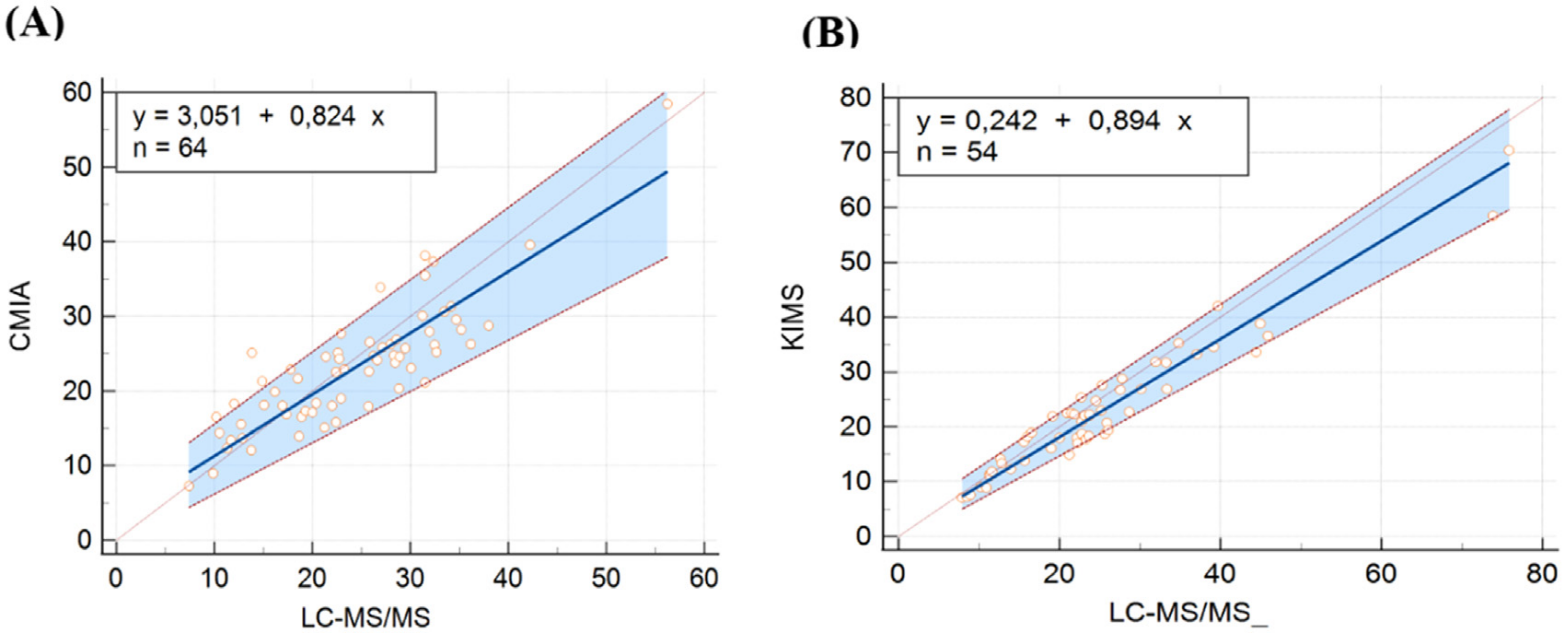
Passing Bablok regression plots comparing vancomycin concentrations measured by (A) LC-MS/MS and CMIA and (B) LC-MS/MS and KIMS. The solid line represents the regression line, the dashed lines represents the confidence interval for the regression line, and the dotted line represents an identity line (*x* = *y*). CMIA, Chemiluminescent Microparticle Assay; KIMS, Kinetic Interaction of Microparticles in Solution; LC-MS/MS, Liquid Chromatography coupled with Tandem Mass Spectrometry.

**Fig. 2:**
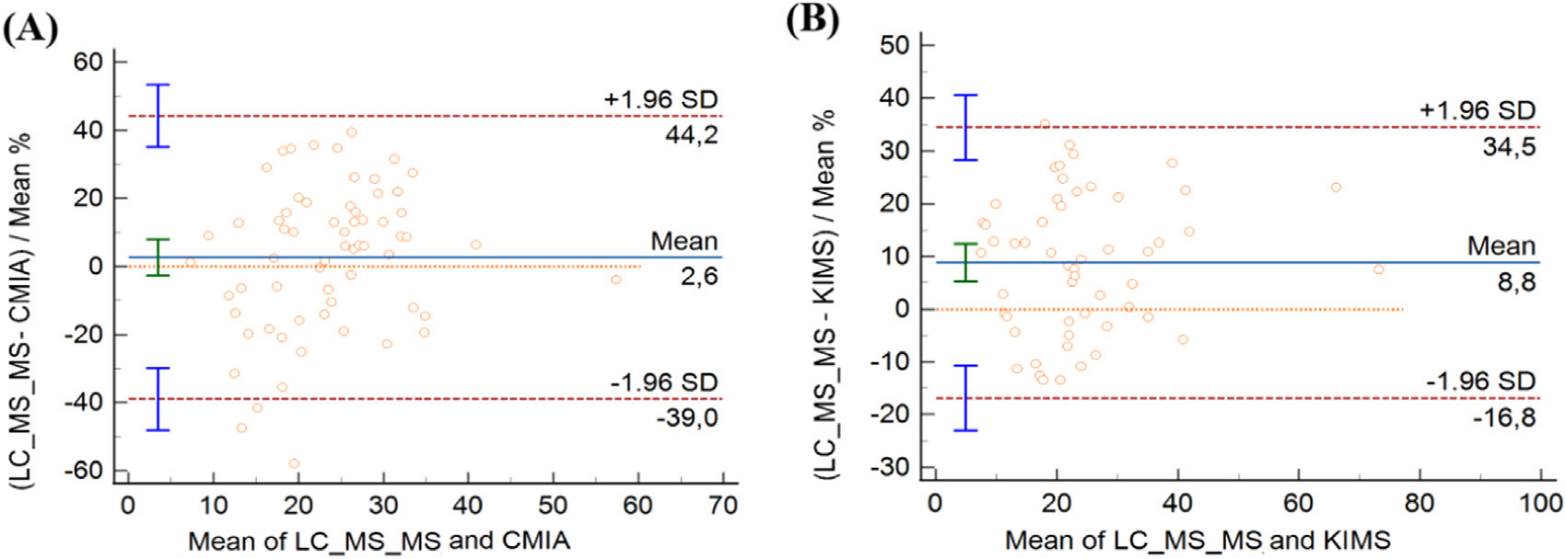
Bland-Altman plots comparing vancomycin concentrations measured by (A) LC-MS/MSCMIA and CMIA and (B) LC-MS/MS and KIMS. CMIA, Chemiluminescent Microparticle Assay; KIMS, Kinetic Interaction of Microparticles in Solution; LC-MS/MS, Liquid Chromatography coupled with Tandem Mass Spectrometry .

In CMIA evaluation, vancomycin concentrations ranged from 7.30 to 58.50 μg/mL with CMIA and from 7.39 to 56.23 μg/mL with LC-MS/MS. CMIA and LC-MS/MS measurements were highly correlated, with a Spearman rank correlation coefficient value of 0.840 (*p* < 0.001, 95 % CI 0.749‒0.900). No deviation of linearity was observed (*p* = 0.81). Regression analysis is shown in Fig. 1A and the results are summarized in Table 2. No systematic bias was observed, as the intercept was not significantly different from 0 μg/mL (95 % CI: −0.6931 to 5.909). Nonetheless, the slope of 0.82 (95 % CI 0.6873‒0.9671) showed a small proportional bias. The mean difference between vancomycin concentrations measured by CMIA and LC-MS/MS was −1.0 μg/mL (−10.38 to 11.26). Bland-Altman plots are shown in Fig. 2A. Most of the differences between both methods were within the ±1.96 standard deviation range. LC-MS/MS measured levels were, on average, 2.64 % higher than CMIA (range: −57.88 % to 39.48 %). The difference between values obtained was within 20 % of the mean for 67 % of the samples (43/64), following ISR.

**Table 2:** Comparison of vancomycin concentrations measured by CMIA and LC-MS/MS.

Evaluation parameter	CMIA cohort (*n* = 64)
Difference of CMIA and LCMS/MS measurements (μg/mL, range)	-1.0 (−10.38 to 11.26)
Passing-Bablok correlation coefficient (95 % CI)	0.840 (0.749 to 0.900)
Passing-Bablok regression intercept (95 % CI)	3.05 (−0.6931 to 5.9097)
Passing-Bablok regression slope (95 % CI)	0.82 (0.6873 to 0.9671)
Cusum (P)	0.81
Bland-Altman mean of differences (95 % CI)	2.64 (−2.6603 to 7.9446)

CMIA, Chemiluminescent Microparticle Assay; LC-MS/MS, Liquid Chromatography coupled with Tandem Mass Spectrometry.

In the KIMS assessment, vancomycin concentrations ranged from 7.10 to 70.40 μg/mL with KIMS and from 7.90 to 75.87 μg/mL with LC-MS/MS. KIMS and LC-MS/MS measurements revealed a high correlation, with Spearman rank correlation coefficient value of 0.926 (*p* < 0.001, 95 % CI 0.875 to 0.957), and no significant deviation of linearity was observed (*p* = 0.49). Fig. 1B represents the regression analysis and the results are summarized in Table 3. The intercept was not significantly different from 0 μg/mL (95 % CI −1.3759 to 2.689), then no systematic bias was observed. The slope was 0.89 (95 % CI 0.8032 to 0.990), representing a small proportional bias. The mean difference between vancomycin concentrations measured by KIMS and LC-MS/MS was −2.28 μg/mL (−15.34 to 2.75). Bland-Altman plots are shown in Fig. 2B. The difference between both methods was within the ±1.96 standard deviation range. LC-MS/MS measured levels were, on average, 8.81 % higher than KIMS (range: −13.40 % to 35.04 %). The difference between values obtained was within 20 % of the mean for 76 % of the samples (41/54).

**Table 3:** Comparison of vancomycin concentrations measured by KIMS and LC-MS/MS.

Evaluation parameter	KIMS cohort (*n* = 54)
Difference of KIMS and LCMS/MS measurements (μg/mL, range)	−2.28 (−15.34 to 2.75)
Passing-Bablok correlation coefficient (95 % CI)	0.926 (0.875 to 0.957)
Passing-Bablok regression intercept (95 % CI)	0.24 (−1.3759 to 2.6898)
Passing-Bablok regression slope (95 % CI)	0.89 (0.8032 to 0.9909)
Cusum (P)	0.49
Bland-Altman mean of differences (95 % CI)	8.81 (5.2370 to 12.3806)

KIMS, Kinetic Interaction of Microparticles in Solution; LC-MS/MS, Liquid Chromatography coupled with Tandem Mass Spectrometry.

## Discussion

Vancomycin TDM for serious MRSA infections in patients undergoing hemodialysis is strongly recommended by the current international consensus.^17^ Given the narrow vancomycin AUC range for therapeutic efficacy and minimal nephrotoxicity, AUC-target is the most accurate and optimal way to guide vancomycin dosing.^5^,^17^ The AUC0–24/MIC is the PK/PD parameter that best describes the vancomycin antimicrobial activity, and a value of AUC0–24/MIC 400‒600 has been shown to be the PK/PD target. Although Bayesian PK models can accurately estimate the AUC0–24 with a single point in the dose interval, and this is the current guidelines’ recommendations for patients not on renal replacement therapy, this is not validated for patients under dialysis. Therefore, current recommendations is still based on a single point evaluation for patients under replacement renal therapy.^17^

Given the relevance of serum concentration monitoring in patients undergoing HD, the vancomycin assay's accuracy must be guaranteed. Differences between methods of vancomycin measuring have been reported in the last years: previous studies reported CDP-1 cross-reaction in different immunoassays, which are the most applied assays at routine laboratories.^8^,^10^,^12^^,^^14^, ^15^, ^16^^,^^21^, ^22^, ^23^, ^24^, ^25^ In addition to CDP-1 cross-reaction, other interferers may also occur, such as impurities present in commercial vancomycin,^26^ immunoglobulin M,^27^ paraproteins, and rheumatoid factor,^28^ dialyzer flow rate^29^,^30^ and the lack of technique standardization between immunoassays.^10^

This study aimed to assess the performance of two different immunoassays for vancomycin measurement compared to LC-MS/MS in patients undergoing HD. As stated earlier, several studies reported the performance of different immunoassays in measuring vancomycin levels. Nevertheless, none of them compared CMIA and KIMS with HPLC-MS/MS specifically in patients receiving HD. For that, 64 samples from 42 patients analyzed by CMIA and 54 samples from 23 patients analyzed by KIMS were included.

Most patients in CMIA and KIMS cohorts were men (67 % and 74 %, respectively), and CRRT was more frequent than IHD in both groups (88 % and 65 %, respectively). Prescribed vancomycin doses every 12 h were more frequent than once a day for CMIA and KIMS cohorts. A large range of concentrations was evaluated by CMIA and KIMS, and measurements were highly correlated with LC-MS/MS, with no deviation of linearity. Both CMIA and KIMS demonstrated acceptable correlation and agreement, although, the slight proportional bias indicates that whereas concentrations increase, differences between immunoassays and LC-MS/MS increase proportionally, but not biased to a clinically significant degree. Therefore, the higher the concentration, the greater the difference between methods. Therefore, although not significant, this accumulation may contribute to the difference between methods. No systematic bias was observed, and the difference between methods was within the ±1.96 standard deviation range. Following ISR, the difference between values was within 20 % of the mean for 67 % of the samples for CMIA and for 76 % for KIMS. However, the range of observed differences (−10.38 to 11.26 and −15.34 to 2.75 for CMIA and KIMS, respectively) seems insufficient to affect clinical decisions on vancomycin dosing.

High-flux membranes present increased surface area and higher permeability, allowing clearance of middle to large molecules, such as vancomycin.^29^,^30^ Since the chemical structure of CDP-1 and vancomycin are very close, dialytic clearance characteristics must be the same.^14^,^30^ As CDP-1 tends to be removed by high-permeability dialyzers, its serum levels should not be sufficient to interfere with vancomycin measurements by immunoassays. The possible removal of CDP-1 by high-permeability dialyzers and the use of monoclonal antibodies may explain the performance of both CMIA and KIMS described in this study.

Our study has some limitations that must be acknowledged. First, although LC-MS/MS method has been validated according to the guidelines proposed by the American FDA for validation of bioanalytical methods,^20^ which includes specificity and sensitivity testing, we do not have a CDP-1 standard to perform analyze the presence of this compound. However, the chromatographic conditions used are similar to those in other studies,^31^,^32^ hence, we expect that interferences will be unlikely. Second, atenolol-d7 was used as IS since there is no availability of a deuterated analogue of vancomycin on the market. Nonetheless, this has unlikely affected our results, since atenolol-d7 is a compound with a similar retention time and will never be present in patient samples. Its use as an internal standard for vancomycin has already also been described in other studies.^32^ Third, we did not test the analyte stability in the matrix. However, −20 °C frozen were analyzed in no more than 15 days, and it has been demonstrated that vancomycin remains stable for up to 6-months at −20 °C.^33^ Finally, although we included a number higher than the minimum recommended of 40 samples, according to CLSI analytical guideline,^18^ a larger sample size could potentially increase the power to detect small but statistically significant differences.

## Conclusion

This is the first evaluation of CMIA and KIMS performance compared to LC-MS/MS in patients undergoing HD. Both CMIA and KIMS showed acceptable correlation and agreement.The small proportional bias noted in our study indicates that whereas concentrations increase, differences between immunoassays and LC-MS/MS increase proportionally. However, the range of observed differences seems not to be of clinical relevance.

Our study suggests that neither CMIA nor KIMS undergo cross-reactivity from CDP-1 for vancomycin measurement in samples obtained from patients undergoing hemodialysis, although CDP-1 concentrations were not measured. The possible removal of this compound by high-permeability dialyzers and the use of monoclonal antibodies may explain the performance of both CMIA and KIMS. In summary, CMIA and KIMS commercial assays provide accurate vancomycin measurements in patients undergoing HD.

## Patient anonymity and informed consent

The study was approved by Institutional Research Ethics Committees. All patients enrolled in the study, or their guardians agreed to participate by a free and informed consent form. The patient's anonymity was ensured.

## Conflicts of interest

The authors declare no conflicts of interest.

## References

[1] Levine D.P. (2006). Vancomycin: a history. Clin Infect Dis.

[2] Butler M.S., Hansford K.A., Blaskovich M.A.T., Halai R., Cooper M.A. (2014). Glycopeptide antibiotics: back to the future. J Antibiot (Tokyo).

[3] Rybak M.J., Le J., Lodise T.P., Levine D.P., Bradley J.S., Liu C. (2020). Therapeutic monitoring of vancomycin for serious methicillin-resistant Staphylococcus aureus infections: a revised consensus guideline and review by the American society of health-system pharmacists, the infectious diseases society of America, the pediatric infectious diseases society, and the society of infectious diseases pharmacists. Clin Infect Dis.

[4] Javorska L., Krcmova L.K., Solichova D., Solich P., Kaska M. (2016). Modern methods for vancomycin determination in biological fluids by methods based on high-performance liquid chromatography ‒ a review. J Sep Sci.

[5] Heil E.L., Claeys K.C., Mynatt R.P., Hopkins T.L., Brade K., Watt I. (2018). Making the change to area under the curve-based vancomycin dosing. Am J Heal Pharm.

[6] Landersdorfer C.B., Nation R.L. (2021). Limitations of antibiotic MIC-based PK-PD metrics: looking back to move forward. Front Pharmacol.

[7] Rybak M.J., Le J., Lodise T.P., Levine D.P., Bradley J.S., Liu C. (2021). Validity of 2020 vancomycin consensus recommendations and further guidance for practical application. Am J Heal Pharm.

[8] Barco S., Castagnola E., Gennai I., Barbagallo L., Loy A., Tripodi G. (2016). Ultra high-performance liquid chromatography-tandem mass spectrometry vs. commercial immunoassay for determination of vancomycin plasma concentration in children. Possible implications for everyday clinical practice. J Chemother.

[9] Vogeser M., Seger C. (2008). A decade of HPLC-MS/MS in the routine clinical laboratory - Goals for further developments. Clin Biochem.

[10] Chen C.-Y., Li M.-Y., Ma L-Y, Zhai X.-Y., Luo D-H, Zhou Y. (2020). Precision and accuracy of commercial assays for vancomycin therapeutic drug monitoring: evaluation based on external quality assessment scheme. J Antimicrob Chemother.

[11] Vitória M.P., Alvarenga C.G.D.S., Vasconcellos Filho L.M., Teixeira Birro J.C., Barbosa M.C., Ferreira M.A.M. (2019). Low serum trough concentrations and high vancomycin minimum inhibitory concentration in methicillin-sensitive staphylococcus aureus from hemodialysis patients in Brazil. Ther Drug Monit.

[12] Somerville A.L., Pharm D., Wright D.H., Pharm D., Rotschafer J.C., Pharm D. (1999). Implications of vancomycin degradation products on therapeutic drug monitoring in patients with end-stage renal disease. Pharmacotherapy.

[13] Hu M.W., Anne L., Forni T., Gottwald K (1990). Measurement of vancomycin in renally impaired patient samples using a new high-performance liquid chromatography method with vitamin B12 internal standard: comparison of high-performance liquid chromatography, emit, and fluorescence polarization immunoassay. Ther Drug Monit Drug Monit.

[14] Kingery J.R., Sowinski K.M., Kraus M.A., Klaunig J.E., Mueller B.A (2000). Vancomycin assay performance in patients with end-stage renal disease receiving hemodialysis. Pharmacotherapy.

[15] Andriguetti N.B., Lisboa L.L., Antunes M.V., Hahn R., Pagnussat L., Hahn S.R. (2019). Comparison of vancomycin serum concentrations measured by chemiluminescent microparticle immunoassay (CMIA) and liquid chromatography-mass spectrometry (LC-MS /MS) in a group of patients with a wide range of creatinine levels. Lat Am J Pharm.

[16] Fan Y., Peng X., Wu H., Liang X., Chen Y., Guo B. (2020). Simultaneous separation and determination of vancomycin and its crystalline degradation products in human serum by ultra high-performance liquid chromatography-tandem mass spectrometry method and its application in therapeutic drug monitoring. J Sep Sci.

[17] Rybak M.J., Le J., Lodise T.P., Levine D.P., Bradley J.S., Liu C. (2020). Therapeutic monitoring of vancomycin: a revised consensus guideline and review of the American society of health-system pharmacists, the infectious diseases society of America, the pediatric infectious diseases society and the society of infectious disease. Am Soc Heal Pharm.

[18] Clinical and Laboratory Standards Institute (CLSI). Measurement Procedure Comparison and Bias Estimation Using Patient Samples; Approved Guideline ‒ Third Edition. CLSI document EP09-A3 (ISBN 1-56238-887-8 [Print]; ISBN 1-56238-888-6 [Electronic]). Clinical and Laboratory Standards Institute, 950 West Valley Road, Suite 2500, Wayne, Pennsylvania 19087 USA, 2013.

[19] Leven C., Padelli M., Chauvet J., Foulquier J.-B., Carré J.-L., Boglione-Kerrien C. (2019). Vancomycin immunoassay: does the Advia Centaur XPT underestimate the exposure of patients? A method comparison study. Clin Chem Lab Med.

[20] FDA F and DA (2018). Bioanalytical method validation Guidance for Industry. Rev Rom Med Lab.

[21] Fan Y., Peng X., Yu J., Liang X., Chen Y., Liu X. (2019). An ultra-performance liquid chromatography-tandem mass spectrometry method to quantify vancomycin in human serum by minimizing the degradation product and matrix interference. Bioanalysis.

[22] Usman M., Hempel G. (2016). Development and validation of an HPLC method for the determination of vancomycin in human plasma and its comparison with an immunoassay (PETINIA). Springerplus.

[23] Tanaka M., Orii T., Gomi T., Kobayashi H., Kanke M., Hirono S. (2002). Clinical estimation of vancomycin measurement method on hemodialysis patient. Yakugaku Zasshi.

[24] Iwamoto T., Kagawa Y., Kojima M. (2005). Factors influencing the overestimation of plasma vancomycin concentrations measured by the Abbott TDx technique. Ther Drug Monit.

[25] Oyaert M., Peersman N., Kieffer D., Deiteren K., Smits A., Allegaert K. (2015). Novel LC-MS/MS method for plasma vancomycin: comparison with immunoassays and clinical impact. Clin Chim Acta.

[26] Cooper A.A., Cowart K., Clayton A., Paul J (2017). Undetectable vancomycin concentrations utlizing a particle-enhanced turbidimetric inhibition immunoassay in a patient with an elevated IgM level. Clin Lab.

[27] Florin L., Vantilborgh A., Pauwels S., Vanwynsberghe T., Vermeersch P., Stove V. (2015). IgM interference in the Abbott iVanco immunoassay: a case report. Clin Chim Acta.

[28] Legatt D.F., Blakney G.B., Higgins T.N., Schnabl K.L., Shalapay C.E., Dias V.C. (2012). The effect of paraproteins and rheumatoid factor on four commercial immunoassays for vancomycin: implications for laboratorians and other health care professionals. Ther Drug Monit.

[29] Pai A.B., Pai M.P. (2004). Vancomycin dosing in high flux hemodialysis: a limited-sampling algorithm. Am J Heal Pharm.

[30] Anandan J.V., Touchette M.A. (1998). Vancomycin clearance with high-flux dialysis membranes. Int J Artif Organs.

[31] Brozmanová H., Kacířová I., Uřinovská R., Šištík P., Grundman M. (2017). New liquid chromatography-tandem mass spectrometry method for routine TDM of vancomycin in patients with both normal and impaired renal functions and comparison with results of polarization fluoroimmunoassay in light of varying creatinine concentrations. Clinica Chimica Acta.

[32] Zhang T., Watson D.G., Azike C., Tettey J.N.A., Stearns A.T., Binning A.R. (2007). Determination of vancomycin in serum by liquid chromatography-high resolution full scan mass spectrometry. J Chromatogr B Analyt Technol Biomed Life Sci.

[33] Halperin S.J., Greenblatt D.J., Prenner J.L., Fine H.F. (2023). Stability of vancomycin and ceftazidime with prolonged storage at −20°C. Ophthalmic Surg Lasers Imaging Retina.

